# Treatment Options for Faecolith (Stercoral) Obstruction of the Colon in Two Similar Cases

**DOI:** 10.7759/cureus.44660

**Published:** 2023-09-04

**Authors:** Philip Holcroft, Faith Solanke, Sana Ullah, Mamoon Solkar, Pardeep Arora

**Affiliations:** 1 General Surgery, Tameside General Hospital, Manchester, GBR; 2 General Surgery, University of Sheffield, Sheffield, GBR

**Keywords:** fecal disimpaction, flexible sigmoidoscopy, emergency laparotomy, faecolith, colon obstruction

## Abstract

A woman and man in their 20s presented to the accident and emergency (A&E) department with abdominal pain, vomiting, and absolute constipation. They both presented as atypical candidates for faecal impaction with few medical and lifestyle risk factors. On CT imaging, a faecolith was visualised in the sigmoid colon as a cause of the large bowel obstruction (LBO) in both patients. A faecolith obstruction can be a life-threatening sequela of faecal impaction. The first line of treatment for LBO is conservative management with oral laxatives and enemas. If this is unsuccessful, interventions such as flexible sigmoidoscopy with the placement of enemas above the blockage can be used. In the event of a compromise to bowel vascularity or if previous interventions prove unsuccessful, there is also scope for surgery. After the resolution of the blockage, follow-up investigations should be performed to elicit an underlying cause in the hope of preventing a recurrence.

## Introduction

Faecal impaction is a common digestive disorder observed in adults. It is where the buildup of faecal matter in the colon, with dehydration and hardening, leads to an inability to evacuate large, hard, concreted stools [1]. In severe cases of faecal impaction, a faecolith may develop. A faecolith is a hardened piece of stool with various compositions that become calcified and appear as an opacity on imaging. They are commonly found to develop in the sigmoid colon or rectum, and their formation can have life-threatening sequelae. If a faecolith causes a complete large bowel obstruction (LBO), the pressure effects from more proximal stool may disrupt bowel vascularity and lead to wall perforation [2]. For this reason, it is important that faecoliths are diagnosed promptly and treated appropriately.

The literature typically presents faecolith formation in the elderly or debilitated, who develop faecal impaction due to long-standing and untreated constipation [2]. In this case report, we describe two unusual but similar presentations of faecolith formation in young, fit, and healthy individuals.

## Case presentation

Case one

A woman in her 20s presented with a two-week history of constipation, left-sided abdominal pain, and vomiting. She also reported a two-day history of absolute constipation with no urinary or gynaecological symptoms. Two years prior, she had a normal laparoscopy to explore ongoing pelvic pain but had no significant medical or surgical history. The patient had no relevant family or social history. On initial examination, she had a soft, distended abdomen with mild left-sided tenderness and no peritonism. She had an empty rectum on the per rectal (PR) examination, and her observations were within normal limits. Her blood work revealed neutrophilic leucocytosis and radiographic imaging was arranged. Intravenous fluids, antibiotics, and anti-emetics were started, and the patient was kept nil by mouth.

**Figure 1:**
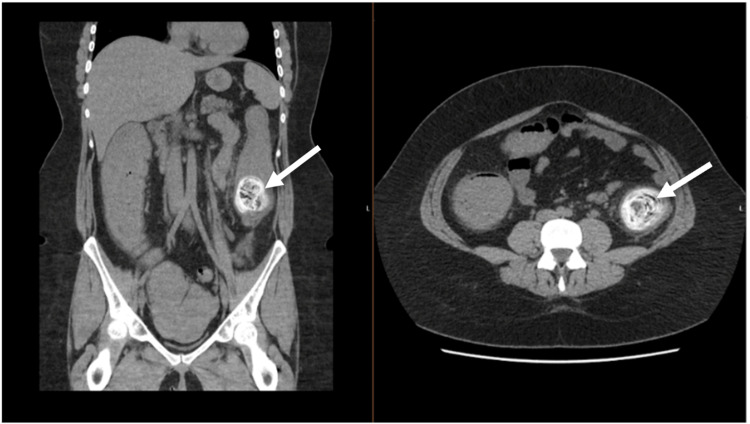
The patient's CT scan (case one) A coronal (left) and transverse (right) CT image of the patient's abdomen and pelvis shows a calcified faecolith in the proximal sigmoid colon.  An initial scan was performed on the patient's admission.

The patient had a plain abdominal radiograph that showed faecal loading. An additional CT scan of the abdomen and pelvis revealed the following: proximal large bowel dilatation and wall thickening, with the right colon dilated to 65mm (Figure 1).

The area within the lumen at the level of the sigmoid colon was of high attenuation with an unusual appearance, most likely a diagnosis of large faecolith, urgent laparotomy was advised.

**Figure 2:**
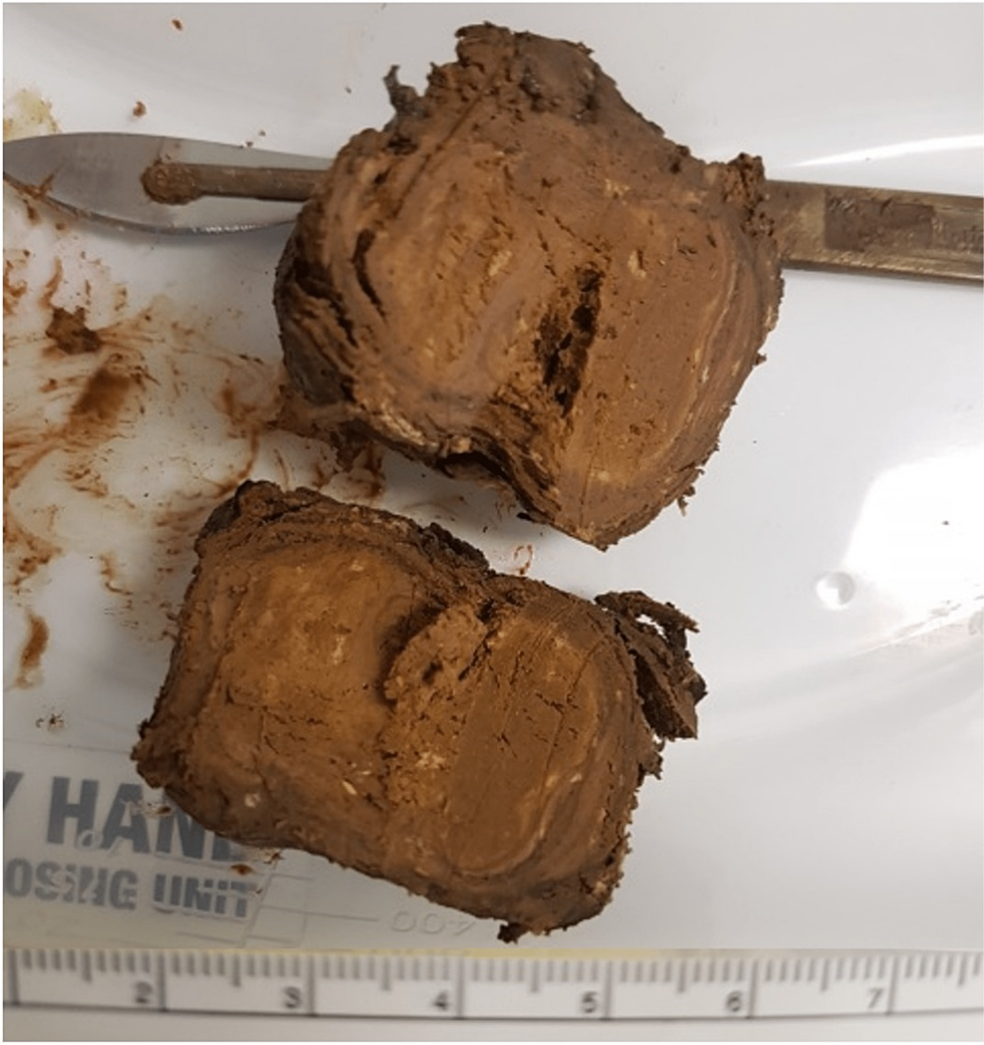
The patient's faecolith (case one) An image of the faecolith removed from the patient's sigmoid colon during an open laparotomy Image courtesy of Mr Pardeep Arora.

A clear provisional diagnosis for this patient was the large faecolith-causing LBO. To relieve the faecolith obstruction, a conservative approach of phosphate enemas and oral laxatives was first used. This therapy was unsuccessful. Next, an attempt with a flexible sigmoidoscopy was made to remove the blockage, with the passage of a phosphate enema through the scope. Again, they were unable to relieve the impaction, and due to excruciating pain, the team decided to perform an emergency diagnostic laparoscopy. During surgery, a grossly distended colon was visualised proximal to the sigmoid faecolith. At the site of faecolith impaction, bowel vascularity was compromised and at risk of perforation. The laparoscopy was turned into an open operation where a transverse incision was made in the left lower quadrant, and the sigmoid colon was brought to the surface and opened, allowing the removal of the faecolith and faecal fluid (Figure 2).

A necrotic patch of the bowel was removed, and a loop colostomy was formed.

The patient stayed in the hospital after surgery for a few days and was discharged with a fully functional stoma. Four months after discharge, she had a reversal of the colostomy, which was successful. There were no complications, and her post-reversal colonoscopy was normal.

Case two

A man in his 20s presented with a three-day history of absolute constipation, non-specific abdominal pain, and vomiting. He previously presented to the hospital with non-specific abdominal pain in April 2021 but was managed conservatively. Otherwise, he had no significant medical history. His surgical history involved an appendicectomy in 2021 for acute appendicitis. On initial examination, he had tenderness in the suprapubic region, with rebound tenderness and localised guarding. He had an empty rectum on the PR examination, and his observations were within normal limits. His blood work revealed neutrophilic leucocytosis and radiographic imaging was arranged. Intravenous fluids, antibiotics, and anti-emetics were commenced, and the patient was kept nil by mouth.

**Figure 3:**
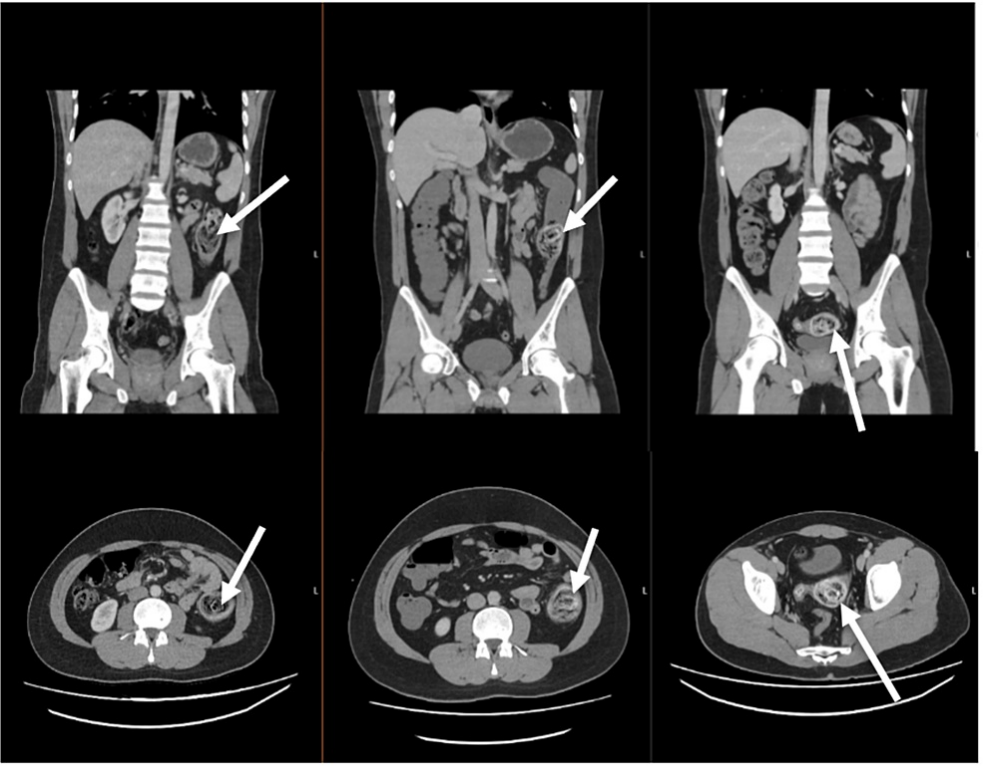
The patient's CT scans (case two) A set of coronal (top row) and transverse (bottom row) CT images of the abdomen and pelvis Images are shown in the order of the patient's presentation: April 2021 (left column), admission June 2023 (middle column), and after flexible sigmoidoscopy June 2023 (right column). The calcified faecolith can be seen migrating from the descending colon (left column) to the proximal sigmoid colon (middle column) and to the distal sigmoid colon (right column).

The patient had a plain abdominal radiograph, which demonstrated a mildly dilated transverse colon and partial obstruction. An additional CT scan of the abdomen and pelvis (Figure 3) revealed the following: a long-standing thickening of the distal half of the descending colon extending to the proximal sigmoid colon, most likely subsequent to long-standing inflammatory changes, and the narrowing of the large bowel with dilatation proximal to this level.

In this patient, the original primary diagnosis was left-sided colitis. However, following a discussion between the radiologist and general surgeon, the provisional diagnosis was revised to faecolith obstruction without compromise to bowel circulation. His faecolith obstruction was initially managed with a conservative approach of phosphate enemas via flexible sigmoidoscopy and oral laxatives. This failed to fully resolve the impaction. A following CT scan showed that the faecolith had migrated from the proximal to the distal sigmoid colon. Two further attempts at flexible sigmoidoscopy with the passage of arachis oil enemas above the obstruction proved partially successful. The faecolith was shown to have migrated to the rectum, as seen on a final plain abdominal radiograph.

Following the third flexible sigmoidoscopy on the patient, the faecolith had moved to his rectum and was visualised on a plain abdominal radiograph. He was able to pass loose stools, and his suprapubic abdominal pain resolved. The patient was discharged with oral laxatives and general lifestyle advice to avoid constipation. He was also given a patient-initiated follow-up appointment that was open access for six months.

## Discussion

There are only a few published articles on faecolith bolus obstruction, with different terminology used within them. Both faecolith and faecoloma, a tumour-like mass, are used interchangeably. This has also been found with stercoral obstruction and faecal impaction [2].

The diagnosis of faecolith LBO can be made after the interpretation of radiological imaging [2]. In case two, the faecolith had not been reported on two occasions after imaging by the previous radiologists. The faecolith was visible in the CT images as an opacity in the mid-descending colon in April 2021 and the proximal sigmoid colon in June 2023 (Figure 3). However, a diagnosis of a faecolith was only made in June 2023 after the general surgeon discussed the images with the radiologist. This case highlights the importance of raising awareness amongst clinicians of how to interpret faecoliths on imaging and remember that they can be a life-threatening cause of LBO.

In faecolith LBO, there are no clear guidelines on the sequence of management. Current literature has described examples of both medical and surgical approaches to management that appear effective in their respective cases. Smith et al. and Atahan et al. both describe a surgical approach to the management of faecolith obstruction [2,3]. They report cases of a 27-year-old woman and a 45-year-old man, respectively, who presented with LBO secondary to a sigmoid faecal bolus. Both cases did not resolve after conservative or endoscopic management and required surgical intervention. In both cases, an emergency laparotomy and transverse colostomy were performed to decompress the colon and remove the impacted faecolith. A case of medical management for faecolith obstruction was presented by Segall H et al., where a 44-year-old man achieved disimpaction of a sigmoid faecoloma with the use of mineral oil enemas [4]. Further cases of medical management include the use of endoscopic Coca-Cola injections to disimpact a sigmoid faecoloma, by both Ontanilla-Clavijo et al. and Lee et al. [5,6]. Key lessons to consider from these literature examples include the need for early and regular imaging in patients with faecal impaction to guide the progression of therapy and the prompt initiation of conservative management for faecal impaction with clinicians regularly reassessing the effects of therapy to decide the escalation of treatment [1,2].

In the present case report, considerations taken by clinicians to determine sequential management were the length of time the patient was in complete bowel obstruction and their subsequent risk of wall perforation. For individuals with extended time in complete obstruction with a high risk of perforation, a surgical approach is more appropriate as it will be faster and more definitive. In cases with a lower risk of perforation, a less invasive medical approach can be utilised.

To prevent the initial occurrence and potential recurrence of faecolith obstruction, it is important to identify the causes of the faecolith formation in the first place [1]. As previously discussed, faecal impaction can be secondary to chronic and untreated constipation. Faecolith formation tends to occur as a severe case in the elderly or debilitated, with poor hydration and reduced mobility [2]. However, this clearly contrasts with the presentation of the two young and healthy individuals demonstrated in the case report. In both cases, they do not have any significant medical or lifestyle factors that would predispose them to a faecolith obstruction. This potentially highlights the need for more investigation into the aetiology of faecolith obstruction. These cases also emphasise the need to increase education, regardless of age and mobility, on health and lifestyle factors such as constipation, frequency of bowel habits, early treatment, and the importance of hydration and mobilisation [1].

## Conclusions

Faecal impaction is a cause of large bowel obstruction, and faecolith formation is a life-threatening sequela of it. Although faecoliths typically present in the elderly or debilitated, they can also present in young and healthy patients with minimal risk factors. Early and regular imaging in patients with faecal impaction is important to guide the progression of therapy. Conservative management should be initiated promptly for faecal impaction, and clinicians should regularly reassess the effects of therapy to decide on the escalation of treatment. On discharge, clinicians should try to elicit the cause of faecal impaction by considering health and lifestyle factors to prevent a recurrence.
